# Treatment of hypoxia‐dependent cardiovascular diseases by myo‐inositol trispyrophosphate (ITPP)‐enhancement of oxygen delivery by red blood cells

**DOI:** 10.1111/jcmm.14909

**Published:** 2020-01-19

**Authors:** Marta Oknińska, Bouchra El‐Hafny‐Rahbi, Aleksandra Paterek, Urszula Mackiewicz, Claire Crola‐Da Silva, Klaudia Brodaczewska, Michał Mączewski, Claudine Kieda

**Affiliations:** ^1^ Department of Clinical Physiology Centre of Postgraduate Medical Education Warsaw Poland; ^2^ Center for Molecular Biophysics UPR 4301 CNRS Orleans France; ^3^ Laboratory of Molecular Oncology and Innovative Therapies MMI Warsaw Poland

**Keywords:** cardiomyocytes, haemoglobin, hypoxia, myocardial infarction, oxygen delivery

## Abstract

Heart failure is a consequence of progression hypoxia‐dependent tissue damages. Therapeutic approaches to restore and/or protect the healthy cardiac tissue have largely failed and remain a major challenge of regenerative medicine. The myo‐inositol trispyrophosphate (ITPP) is a modifier of haemoglobin which enters the red blood cells and modifies the haemoglobin properties, allowing for easier and better delivery of oxygen by the blood. Here, we show that this treatment approach in an in vivo model of myocardial infarction (MI) results in an efficient protection from heart failure, and we demonstrate the recovery effect on post‐MI left ventricular remodelling in the rat model. Cultured cardiomyocytes used to study the molecular mechanism of action of ITPP in vitro displayed the fast stimulation of HIF‐1 upon hypoxic conditions. HIF‐1 overexpression was prevented by ITPP when incorporated into red blood cells applied in a model of blood‐perfused cardiomyocytes coupling the dynamic shear stress effect to the enhanced O_2_ supply by modification of haemoglobin ability to release O_2_ in hypoxia. ITPP treatment appears a breakthrough strategy for the efficient and safe treatment of hypoxia‐ or ischaemia‐induced injury of cardiac tissue.

## 1 INTRODUCTION

Heart failure, especially with reduced ejection fraction, is a complex syndrome characterized by ongoing progression of the disease without any external triggering factors.^1^ Cardiac structure and function continue to deteriorate.^2^ This progression is believed to be powered by multiple vicious circles that eventually result in end‐stage pump failure and patient's death.^1^ They include cardiomyocyte apoptosis and necrosis, resulting in net loss of cardiomyocytes, mitochondrial dysfunction, impaired cardiomyocytes Ca^2+^ handling, fibrosis, neurohormonal activation and ischaemia.^2^ Currently, heart failure therapy is aimed at inhibiting detrimental neurohormonal activation (ACE inhibitors, sartans, beta blockers) and is able to merely slow down the progression of the disease rather than stop it or preferably reverse it.

Heart failure is emerging as an energy starvation state, that is cardiomyocytes are ATP depleted.^3^ Studies suggest that intracellular ATP concentration may be 25%‐30% lower in the failing human hearts^4^ and this process can contribute to heart failure progression. This is probably a multifactorial phenomenon, resulting from reduced oxygen delivery, impaired oxygen utilization in the face of increased oxygen demand (due to increased afterload).^5^ So far therapeutic attempts to address it have largely been unsuccessful. They were mainly focused on increasing the concentration of haemoglobin, an oxygen‐carrying protein. But neither erythropoietin analogs that increased haemoglobin concentration^6^ nor intravenous iron that provided an essential element not only for haemoglobin, but also other enzymes involved in cardiac energetics^7^ provided unequivocal benefits in human clinical trials, though recent data, including our own work,^8^ suggest that iron may be of some value here.

Another drastically different approach involves facilitation of oxygen dissociation from haemoglobin in the tissues. Only 25% of oxygen transported by haemoglobin (Hb) dissociates and is retained in tissues, while the remaining three quarters return with venous blood. Under low tissue oxygen content, the extraction rate increases. Endogenous allosteric regulators (2,3‐bisphospho‐d‐glycerate (BPG)) bind to the allosteric cavity of Hb, stabilizing the deoxygenated form of Hb, and oxygen affinity is reduced favouring further oxygen dissociation and retention by tissues. Oxygen extraction rate increases even further, but never exceeds 75%. So one‐quarter of oxygen is returned with venous blood being a potential target for intervention. In this regard, *myo‐inositol trispyrophosphate* (ITPP), a membrane‐permeant allosteric effector of haemoglobin, has been shown to enhance the oxygen release capacity of red blood cells^9^ by lowering the affinity of haemoglobin for oxygen, thus enabling to counteract the effects of hypoxia.^10^ Indeed, we have previously shown that ITPP shifts the oxygen dissociation curve downward, therefore increases tissue oxygen delivery, especially to hypoxic tumours.^11^ This is accompanied by a potent anti‐cancer effect in various tumour models in the mouse.^11^ Thus, ITPP anti‐cancer effects in animal models involve better oxygen delivery to the tumour as part of the vessel normalization process which is the key to the effectiveness of the treatment*.*

Hypoxia‐inducible factor (HIF) is emerging as the main mediator of hypoxia and ischaemia. Highly conserved, it plays a pivotal role in the transcriptional response to changes in oxygen availability. HIF‐1 comprises two subunits, the α‐subunit, which is regulated in an oxygen‐dependent manner and β‐subunit that is constitutively expressed. Molecular oxygen allows for HIF‐1α hydroxylation by prolyl hydroxylase 2 (PHD2) and its rapid proteosomal degradation after von Hippel‐Lindau protein binding and ubiquitinylation. On the other hand, hypoxia impairs hydroxylation, which results in HIF‐1α stabilization, heterodimerization with HIF‐1β. The heterodimer (HIF‐1) enters the nucleus and binds to the hypoxia response element (HRE) to start the transcription of the subsequent hypoxia‐inducible genes.^12^ Thus, HIF‐1 controls the expression of multiple genes that regulate cell survival, cell metabolism and angiogenesis in hypoxic conditions.^13^, ^14^ In a short term, HIF‐1 activation can be beneficial by switching the cardiac metabolism to anaerobic pathway, reducing energy consumption by down‐regulating Ca^2+^ handling proteins (SERCA2a) that utilize enormous quantities of ATP and stimulating angiogenesis. However, long‐term HIF‐1 activation can be detrimental, by inducing energy starvation through reduced fatty acid oxidation and impaired mitochondrial biogenesis^15^ and impairing Ca^2+^ handling. Furthermore, we have recently shown that angiogenesis stimulated by HIF‐1 in tumour results in the formation of defective vessels that are leaky and do not provide adequate blood supply.^11^ In this context, we have demonstrated that ITPP reduces HIF‐1α expression by vascular endothelial cells,^16^ providing anti‐tumour effects.^17^ Although the extent and dynamics of HIF‐1 expression in hypoxic tumour cells as well as its response to ITPP have been studied in details, behaviour of this system in cardiomyocytes in the context of ischaemic cardiac diseases is largely unknown as well as the effect of ITPP in their treatment.

Thus, the first aim of our study was to test the hypothesis that ITPP has beneficial effect on post‐MI left ventricular remodelling and heart failure in the rat model. The second aim was to verify the hypothesis that HIF‐1 expression is rapidly stimulated by hypoxia and that this HIF‐1 overexpression is prevented by ITPP in a model of murine blood‐perfused cardiomyocytes.

## 2 MATERIALS AND METHODS

Male Wistar rats (n = 35) 280‐320 g and female C57Bl6 mice were used in this project. All animal procedures conformed to the guidelines from Directive 2010/63/EU of the European Parliament on the protection of animals used for scientific purposes. The study was approved by the local ethics committee (Second Warsaw Local Ethics Committee for Animal Experimentation).

### 2.1 Heart failure in vivo rat study—experimental design

**Figure 1:**
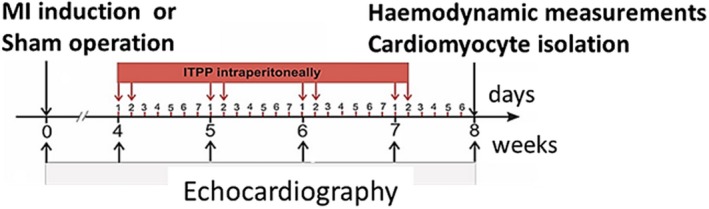
Experimental protocol. MI‐myocardial infarction; ITPP‐*myo‐inositol trispyrophosphate*

Rats were subjected to induction of myocardial infarction (MI group) or sham operation (Sh group). After 4 weeks of follow‐up, the animals underwent echocardiographic imaging. Only rats with large MI (≥40% of the LV) were enrolled to MI group in this study (5 rats were excluded). Both MI and Sh rats were randomized into two subgroups: ITPP group received 2 intraperitoneal doses of ITPP (1.5 g/kg) per week (on Monday and Tuesday), a total of eight doses over 4 weeks (see Figure 1 for study protocol), while saline group received corresponding saline injections. We have previously shown in a mouse model that such dosing regimen provides long‐term improvement of oxygen delivery to the tumour and survival benefits.^11^ Echocardiography was performed weekly. Eight weeks after MI induction/sham operation, the rats underwent final echocardiographic imaging and ketamine HCl/xylazine overdose was given to euthanize the animals and subsequently hearts were excised for biochemical and cellular studies.

#### 2.1.1 Induction of myocardial infarction

Rats were anaesthetized with ketamine HCl and xylazine (100 mg/kg and 5 mg/kg b.wt., respectively, ip), left thoracotomy was performed, the heart was externalized, and a suture (5‐0 silk) was placed around proximal left coronary artery. In Sh animals, it was left loose, and in MI animals, it was tied. Buprenorphine (1 mg/kg b.wt., ip) was given as a post‐operative analgesia. Within 4 weeks, post‐MI mortality was 40%. The vast majority of these deaths (18 animals) occurred during first 2 days after induction of MI. No death occurred after randomization to ITPP/saline groups 4 weeks after MI induction and completion of the study.

#### 2.1.2 Echocardiography imaging

Echocardiography was performed using SonoScape S2 (Sonoscape Medical) with high‐frequency linear 8 to 15 MHz probe. Each rat was examined 4, 5, 6, 7 and 8 weeks after the surgery. Under light isoflurane anaesthesia, LV end‐diastolic and end‐systolic diameters were determined from the long‐axis view at the aortic valve level. Regional LV wall motion abnormalities were assessed using the wall motion index (WMI). Contractility of 12 wall segments visualized in the midpapillary short‐axis view and 11 segments visualized in the long‐axis view was graded as 1 (normal) or 0 (abnormal), and the total WMI was calculated (Figure 1C). The normal hearts had WMI = 23. Our previous results revealed that WMI closely correlated with infarct size and that WMI = 15 corresponded to infarct size ~40%. LV ejection fraction (LVEF) was calculated as (LV diastolic area—LV systolic area)/LV diastolic area. All measurements were obtained by one observer blinded to the study groups.

#### 2.1.3 Myocyte isolation

After excision of the heart, the aorta was cannulated and retrogradely perfused for 5 minutes with nominally Ca^2+^‐free solution containing 100 μmol/L of EGTA of the following composition (in mmol/L): 144 NaCl, 5 KCl, 1 MgCl2, 0.43 NaH2PO4,10 N‐2‐hydroxyethylpiperazine‐N˘‐2‐ethanesulfonic acid (HEPES), 11 glucose and 5 sodium pyruvate. The pH of the solution was adjusted with NaOH to 7.3 Initial washout period was followed by 20 minutes of perfusion with Ca^2+^ free Tyrode solution containing 20 mg collagenase B (Roche) and 3 mg protease (Sigma) per 30 mL. Thereafter, the right ventricle was separated and discarded. LV was placed in the glass dish and mechanically disrupted. The cell suspension was filtered and allowed to sediment. The supernatant was discarded, and cells were washed twice with Tyrode's solution, the Ca^2+^ concentration being increased gradually to 1 mmol/L. The myocytes were superfused at 37°C with Tyrode's solution containing 1.8 mmol/l Ca^2+^.

#### 2.1.4 Myocyte contractions recording

The myocytes contractions were recorded by a video‐edge detection system including a fast digital dimensioning video camera (IonOptix LLC, Milton USA) enabling acquisition of the cell and sarcomere length changes in the real time. The myocytes contractions were elicited by electrical pacing at 1Hz. Sarcomere shortening was taken as a measure of myocyte contractile performance due to more stable registrations at sarcomere than at myocyte level. Amplitude and time‐course of the signal were analysed by the IonWizard software (IonOptix). Amplitude of cell shortening was expressed as the difference between systolic and diastolic sarcomere length and normalized as a percentage of the resting sarcomere length. The contraction time (time to peak) was calculated as the time from the initiation of contraction to the maximal sarcomere shortening. The time required for re‐lengthening sarcomere to the 90% of resting sarcomere length was taken as the relaxation time. The rate of cardiomyocyte contraction and relaxation was calculated for each cardiomyocyte.

### 2.2 Isolated cell experiments

#### 2.2.1 Cells isolation and cell lines establishment

Cardiomyocytes from newborn mice (1 day) and rats (2 days) were obtained upon tissue dilaceration, resuspension and plating for 2 hours in OptiMEM (Gibco, France). Adhering cells on gelatin‐coated Petri dishes were further grown as primary cultures. To test the ability of ITPP to increase RBC oxygen delivery in vitro*,* cardiomyocytes were immortalized according to our previously described method patented for cell keeping a stable phenotype (CNRS patent number 99‐169, world patent N13/521715). Three clones were obtained from rat cardiomyocytic cell primary cultures RCmCl.4, RCmCl.13 and RCmCl.18, and three clones were selected MCmCl.4, MCmCl.10 and MCmCl.14.^17^ After characterization and sorting of the cells that are properly expressing cardiomyocytic markers: myosin, desmin and α‐actinin (anti‐myosin heavy chain Fluorescein‐conjugated Antibody, anti‐Hu/Mo desmin Goat IgG1 and antimouse α actinin IgG2‐FITC labelled, were from R and D) and displayed contractility, the cells were cloned by flow cytometry cell sorting. Mouse derived clone 4 was selected and multiplied (MCmCl.4).

#### 2.2.2 HIF‐1α production by cardiomyocytes submitted to hypoxia compared to normoxia

##### ELISA quantification HIF‐1α production by cardiomyocytes

Cells were subjected to hypoxia pO_2_ = 7.6 Torr = 1%, for various time lapses (from 1 to 12 hours) then lysed, and total HIF‐1α was measured by ELISA (mouse total HIF‐1α kit, R&D systems).^18^

##### HIF‐1α induction, distribution and dynamics of redistribution in cardiomyocytes upon hypoxia treatment

Murine cardiomyocytes were hypoxia‐treated (1% O_2_) for 1‐12 hours, and HIF‐1α induction was detected by anti‐HIF‐1α antibodies (R and D), evidenced by FITC‐labelled secondary antibodies and assessed by fluorescence microscopy (Zeiss axiovert 200M); nuclei were labelled by DAPI (UV and blue light detection). Image analysis for the cellular distribution was done using the Axiovision program.

#### 2.2.3 In vitro* design of RBC‐ITPP effect of oxygen release on cardiomyocytes*

Cardiomyocytes were cultured on microscope slides quadriderm (Dutcher dish) previously coated with 300 µL of 0.2% gelatine aqueous solution, in sterile conditions then dried. Cardiomyocytes (5.10^5^ cells in 3 mL of complete DMEM (10% SVF + 1% Penicillin‐streptomycin + 0.1% Fungizone) were seeded and grown for two days at 37°C in 5% CO_2_ atmosphere. For flow experiments, cultures were submitted to hypoxia (O_2_, 1%) for 3 hours then further treated for 2 hours, by medium maintained either in hypoxia or in normoxia, either containing ITPP‐charged (mRBCs+) or non‐treated (mRBCs−) murine red blood cells. 50 μM ITPP and IHP (inositol hexaphosphate) solutions were used as controls. The role of the shear stress and flow were assessed by flowing the medium containing or not mouse erythrocytes (10^7^ cells/mL) that have been previously charged by ITPP (mRBC+) comparatively to native erythrocytes (mRBC−).

Controls were obtained by the same treatments in normoxia as well as treatment with normal culture medium in flow conditions. When indicated, cells were first incubated for 3 hours in hypoxia and then submitted to flow and maintained for 2 hours.

#### 2.2.4 ITPP‐mRBC loading

Mouse erythrocytes were loaded by addition of a 78 mmol/L ITPP solution in saline to mRBC suspension (1.2 × 10^10^ cells/mL) and 1 hour incubation at 37°C. Washed cells were further used at 10^7^ cells/mL in the appropriate medium for the flow experiments.

#### 2.2.5 ITPP‐mRBC mediated suppression of hypoxia‐induced molecules in cardiomyocytes in vitro

Murine clone 4 cells, selected and allowed to multiply (MCmCl4), were subjected to hypoxia PO_2_ = 7.6 Torr = 1%; for various time lapses (from 1 to 12 hours) and tested for HIF‐1α expression.^18^

### 2.3 Statistical analysis

All data are expressed as means ± SEM. Normal data distribution was verified by Shapiro‐Wilk test while homogeneity of variances by Bartlett's test. Differences among groups were tested by one‐way analysis of variance with a Tukey test as post hoc test or Kruskal‐Wallis test with a post hoc Dunnett‐Tukey‐Kramer test (R version 3.2.0). *P* < .05 was accepted as a level of significance.

In vitro cell, data are expressed as means ± SEM. All statistical analyses were performed using GraphPad Prism 7.0 software.

*P* values were determined by Student's *t* test. A *P* value of less than 0.05 was considered statistically significant. *Indicates statistically significant differences (*P* < .05).

## 3 RESULTS

### 3.1 Heart failure in vivo rat study

#### 3.1.1 Infarct size, morphological parameters and mortality

Rats were subjected to induction of MI or sham (Sh) operation, followed for 4 weeks so that MI rats could develop stable heart failure and then treated for another 4 weeks with either ITPP or saline (Figure 1). The mean infarct size evaluated by transthoracic echocardiography did not differ between MI and MI + ITPP groups at randomization or at the end of the study (4 and 8 weeks after MI induction, respectively) (Table 1). There were no differences in body weight between MI and Sh animals at any time point after the surgery. No death occurred in either study group after beginning of ITPP/saline injections.

**Table 1:** The effect of ITPP on the morphometric and haemodynamic parameters in sham operated and post‐myocardial infarction rats

	Sh (n = 6)	Sh + ITPP (n = 6)	MI (n = 6)	MI + ITPP (n = 6)
Infarct size at 4 wk (%)	‐	‐	47.11 ± 2.43	48.62 ± 4.17
Infarct size at 8 wk (%)	‐	‐	48.30 ± 3.29	47.22 ± 3.38
Body weight (g)	410 ± 12	402 ± 17	411 ± 20	415 ± 28

Note

Means ± SEM.

Abbreviations: BW, body weight; MI, myocardial infarction; Sh, sham operation.

#### 3.1.2 Haemodynamic function and left ventricular dimensions

**Figure 2:**
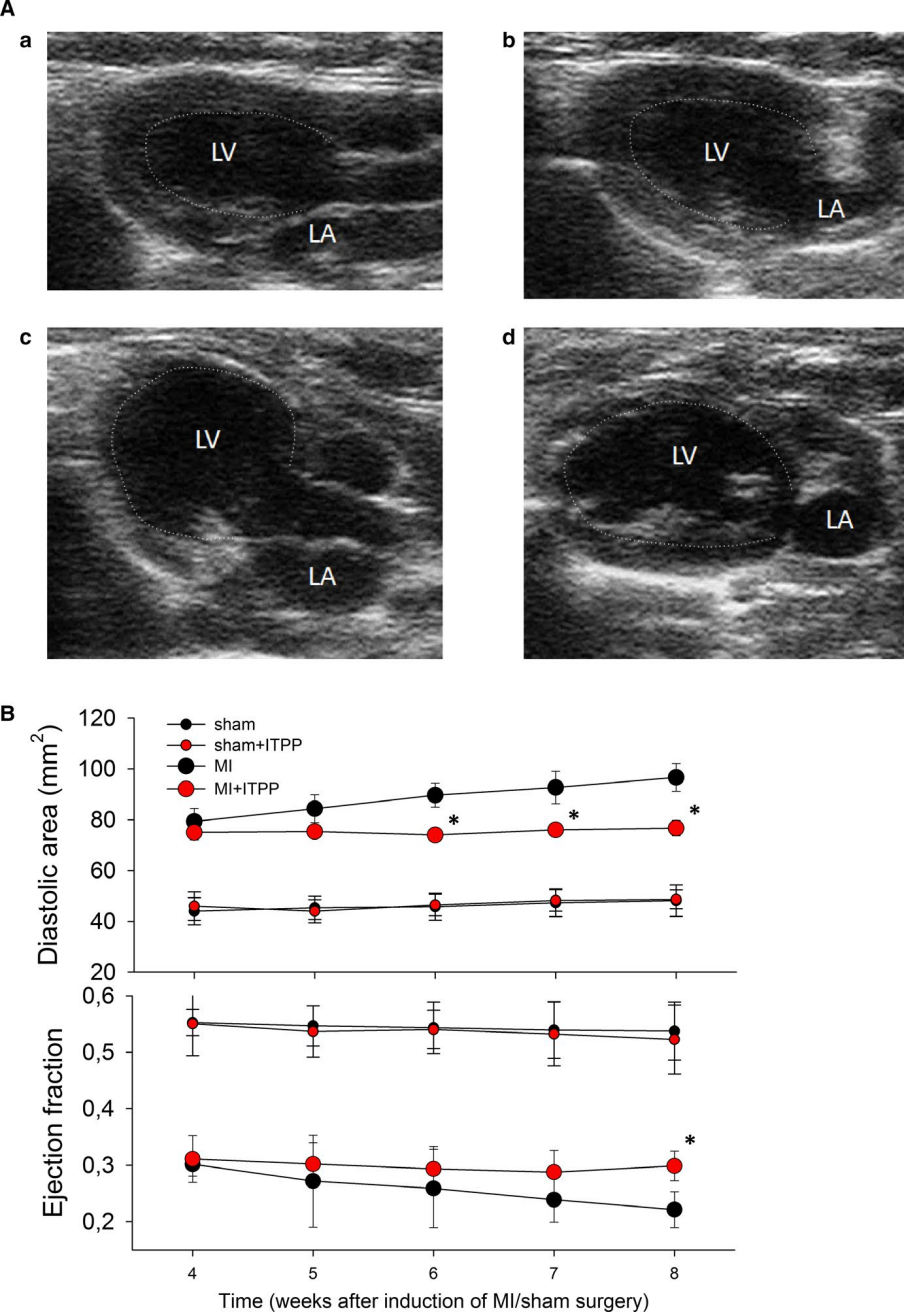
The effect of ITPP on echocardiographic parameters in sham operated (Sh) and myocardial infarction (MI) rats. A, The representative echocardiographic images of LV end‐diastolic area from the long‐axis parasternal view. (a) Sh; (b) Sh + ITPP; (c) MI; (d) MI + ITPP. LV, left ventricle; LA, left atrium. Dashed line marks the endocardial surface. While in both sham images the LV cavity is oblong in shape, it is almost spherical in MI image; B. Left ventricular diastolic area (upper panel) and ejection fraction (lower panel). ^*^ *P* < .05 MI + ITPP vs MI

Large myocardial infarction induced LV remodelling and impaired LV systolic function, reflected by progressive increase of LV end‐diastolic volume and decrease of LV ejection fraction (Figure 2). ITPP essentially halted the increase of LV dilation and significantly reduced impairment of LV ejection fraction, while it had no effect on sham‐operated rats (Figure 2).

#### 3.1.3 Contractile function of cardiomyocytes isolated from the rat left ventricle

**Figure 3:**
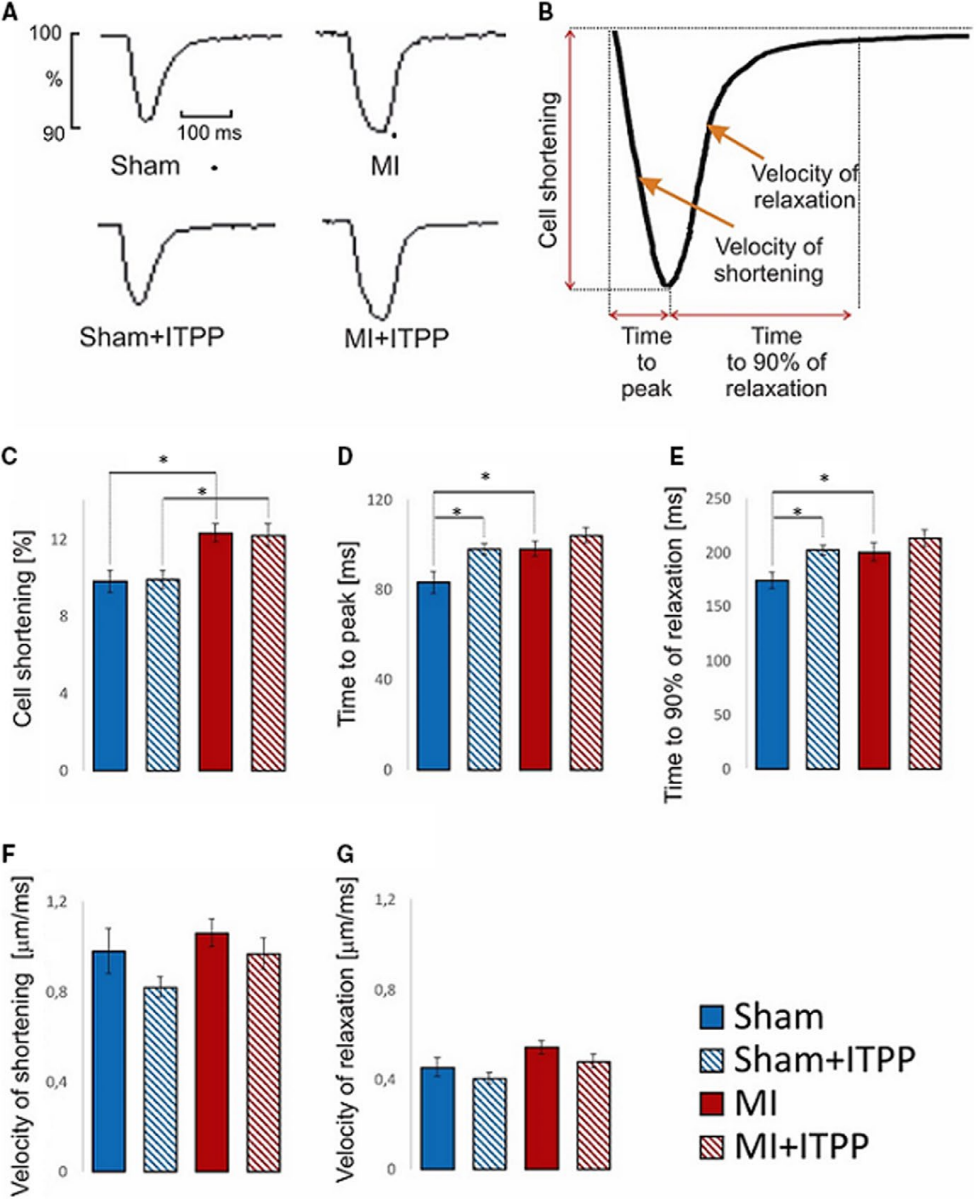
Effect of ITPP on the cardiomyocyte function after myocardial infarction or sham operation. A, representative recordings of contraction in LV cardiomyocytes isolated from sham or infarcted (MI) rats treated or untreated by ITPP. B, schematic presentation of parameters describing cardiomyocyte contraction. Cell shortening (C), time to peak (D), time to 90% of relaxation (E), contraction (F) and relaxation (G) velocities measured in LV cardiomyocytes isolated from rats 8 wk after sham operation or MI induction treated by ITPP or saline. n = 33‐55 cardiomyocytes in each group isolated from at least 3 rats in each group. Only significant differences are shown (^*^ *P* < .05)

Eight weeks after induction of MI and five days after the last dose of ITPP, contractile performance of freshly isolated cardiomyocytes was evaluated (Figure 3A and B). We found that contractile function of LV cardiomyocytes surviving after MI was preserved. Moreover, the amplitude of the cell shortening was even slightly increased (Figure 3C). This is probably a result of the adaptive process of the cardiomyocytes surviving MI to sustain left ventricular function after the loss of numerous cardiomyocytes. As a result of increased amplitude of cell shortening, both time to peak of contraction and time to 90% of relaxation were longer as compared to these parameters in cardiomyocytes from sham‐operated rats (Figure 3D and E, respectively). The rates of cardiomyocyte contraction and relaxation were not changed (Figure 3F and G, respectively).

ITPP administration after MI did not affect cardiomyocytes function and did not affect their functional remodelling. On the other hand, in sham‐operated rats ITPP slightly prolonged the time of contraction and relaxation (Figure 3D and E) and showed a trend towards slower kinetics; however, it was statistically insignificant (Figure 3F and G).

### 3.2 Cardiomyocytic cell line experiments

#### 3.2.1 HIF‐1α is induced, stably expressed and redistributes in cardiomyocytes upon hypoxia treatment

**Figure 4:**
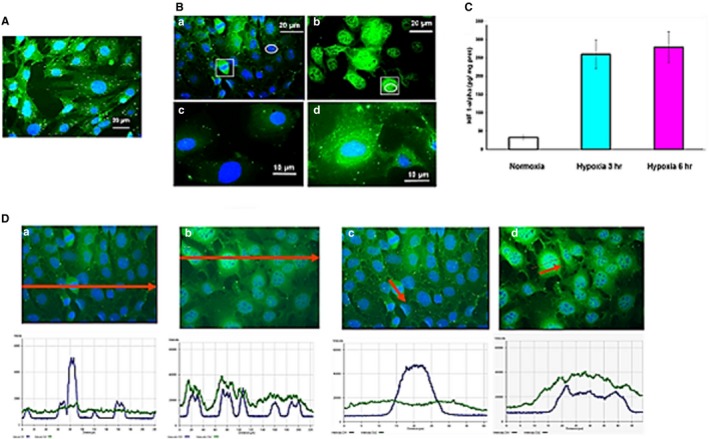
Cardiomyocytic cells characterization and response to ITPP‐modified erythrocytes (RBC‐ITPP^+^). A, Cardiomyocytes MCmCl4 labelling for FTC anti‐α‐actinin antibodies. B, Cardiomyocytes expression of HIF‐1α upon hypoxia treatment. (a) Normoxia control, (b) Hypoxia 1% for 3 hours; (c) control; (d) Hypoxia 1% for 12 hours. C, HIF‐1α kinetics of production by cardiomyocytes upon hypoxia (1%) measured by ELISA. Data represent the mean values ± SEM from experiments performed 4 times (N = 4) in triplicate (n = 3). D, Distribution of HIF‐1α in cardiomyocytes and colocalization with nuclei upon hypoxia (1%) treatment. (a, c) Normoxic controls; (b, d) hypoxia 1%, for 3 hours. Nuclei are labelled by DAPI, and HIF‐1α was labelled by FTC anti‐HIF‐1α antibodies. Microscope is Zeiss Axiovert video microscope equipped with Apotome

Cardiomyocytic cells from newborn mice, established as non‐transformed immortalized line, were submitted to flow cytometry cloning. Clones were characterized for the expression of cardiomyocytic markers as myosin, desmin and α‐actinin. Figure 4 shows the expression of α‐actinin by cloned MCmCl.4 cells (Figure 4A).

When submitted to hypoxia treatment (pO_2_ = 1%) for 3 hours, cardiomyocytes displayed induced HIF‐1α expression (Figure 4B, b) as compared to control cells (Figure 4B, a) together with a redistribution of its localization from the cytosol to the nucleus and a clear increase of the size of the cell nuclei (Figure 4B a,b insets). Upon hypoxia treatment for 12 hours as shown on Figure 4B, d compared to the control on Figure 4B, c, there was accumulation of HIF‐1α in the perinuclear cytosol and the nuclei. The kinetics of HIF‐1α synthesis and production by cardiomyocytes under hypoxia (Figure 4C) indicates that HIF‐1α accumulated actively during the first 3 hours in hypoxia and reached a plateau by 6 hours incubation and then decreased after 12 hours (data not shown).

HIF‐1α induction shown by confocal microscopy analysis in cardiomyocytes upon hypoxia treatment for 3 hours (Figure 4C) was stable for 12 hours under hypoxia. HIF‐1α redistributed from the cytosol to the nucleus as shown by image analysis of the fluorescence distribution of FTC anti‐HIF‐1α as compared to the DAPI as nuclei specific tracer. Figure 4D displays the detection of HIF‐1α basically expressed in the cytosol of cardiomyocytes in normoxia. No colocalization with the nuclei was observed when cells were in normoxia (Figure 4D a and c) but induced HIF‐1α colocalized with the nuclei when cells were treated for 3 hours by hypoxia (1%) (Figure 4D b and d).

The quantification of the fluorescence distribution for DAPI compared to fluorescein (HIF‐1α) at the one‐cell level (Figure 4D c and d, arrows) and the whole population (Figure 4D a and b, arrows) show that upon hypoxia the average size of the nuclei was doubled (Figure 4D, b compared to Figure 4D, a) and the HIF‐1α was detected both in the nucleus as well as in the perinuclear region of the cytosol (Figure 4D a, arrow).

#### 3.2.2 ITPP‐charged murine erythrocytes reduce HIF‐1α expression in hypoxia‐treated cardiomyocytes under flow conditions

**Figure 5:**
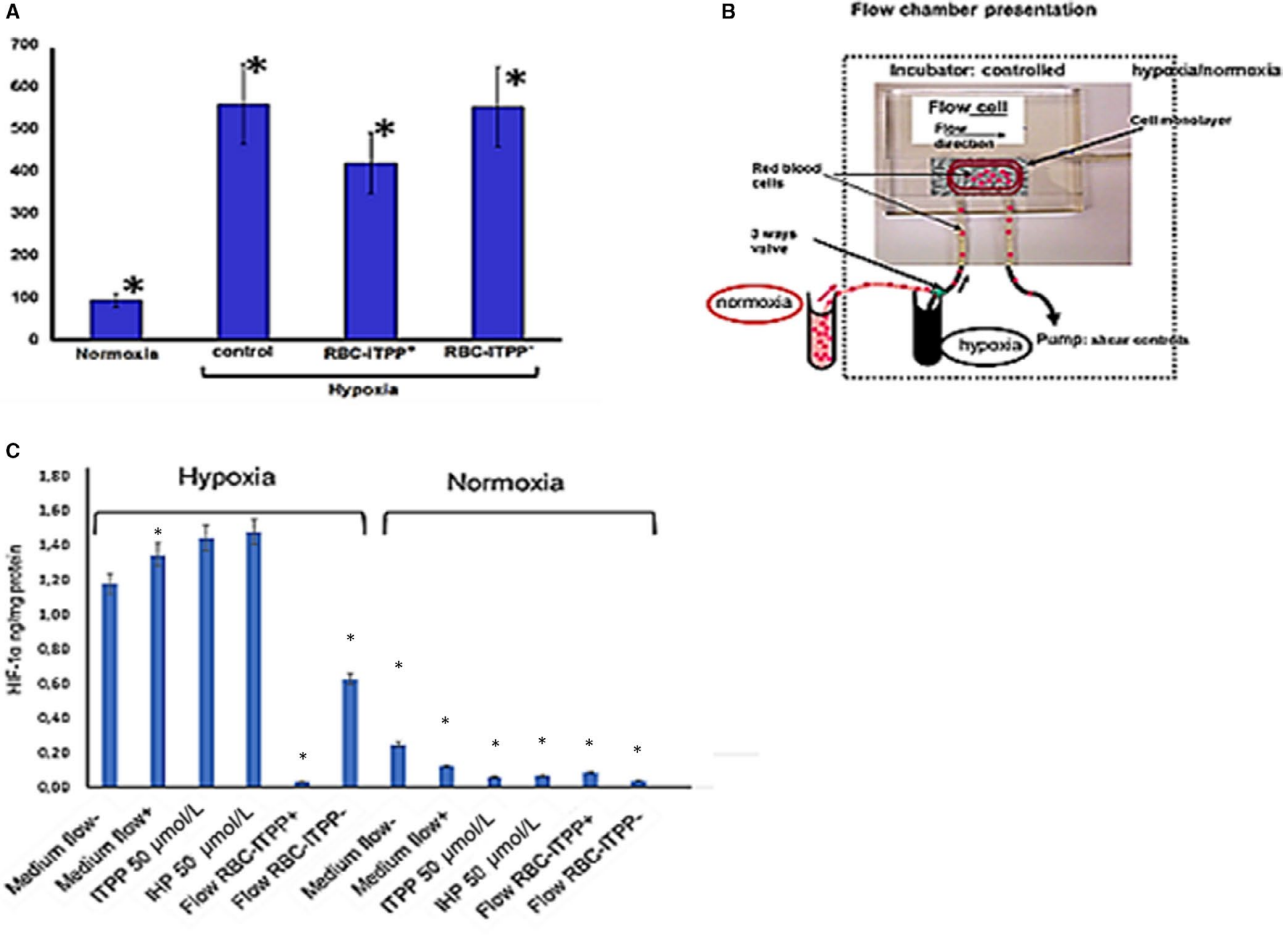
HIF‐1α in cardiomyocytes is reduced by ITPP‐charged red blood cells (RBC‐ITPP^+^)‐ mediated oxygen delivery in flow conditions. A, Effect of RBC‐ITPP^+^ on the modulation of HIF‐1α expression in hypoxia, as compared to RBC‐ITPP^−^. HIF‐1α was detected by ELISA, experiments were performed in triplicates. Data represent the mean values ± SEM from four experiments, in triplicates. B, Flow chamber design for the in flow modulation of a cardiomyocytic cell culture. C, Modulation of cardiomyocytes production of HIF‐1α under hypoxia by flow borne ITPP‐charged RBC‐ITPP^+^ as compared to non‐modified RBC‐ITPP^−^ erythrocytes. Controls are performed in the presence of medium with and without applied flow, and in the presence of ITPP (50 μmol/L) or IHP (50 μmol/L). Data represent the mean values ± SEM from three experiments in triplicates

Cardiomyocyte cultures express HIF‐1α when submitted to hypoxia (O_2_ 1%) for 3 hours. To test the ability of ITPP‐charged red blood cells to deliver oxygen and alleviate hypoxia, cardiomyocytes were perfused with media containing mouse erythrocytes either native, as control, or previously charged with ITPP, as described.^19^ Cardiomyocytes were further treated for 2 hours, in various conditions by medium containing mouse erythrocytes 10^7^cells/mL that were previously charged by ITPP (RBC‐ITPP^+^) compared to non‐charged native erythrocytes (RBC‐ITPP^−^). Figure 5A indicates that HIF‐1α, induced by hypoxia was not modified by the addition of non‐modified red blood cells, while a HIF‐1α production was significantly reduced by 30% after 2 hours of treatment by RBC‐ITPP^+^.

As the effect of the blood‐borne molecules and cells delivery is linked to the dynamics and the shear stress effect, we tested the effect of ITPP‐charged erythrocytes on hypoxic cardiomyocytes in flow conditions. We designed and used a sealed flow chamber which allows the controlled flow and gas conditions as shown on Figure 5B. As summarized on Figure 5C, under normoxic conditions, HIF‐1α protein was expressed at a low level. This was not significantly modified by the control medium or by the same medium containing either the native mouse red blood cells or the ITPP‐loaded mouse red blood cells. Under flow, hypoxia caused a marked induction of HIF‐1α expression as compared to hypoxia without shear stress effect. This is confirmed by the increased effect observed when the flow was applied with medium containing red blood cells as compared to RBC‐ITPP + action in static conditions (Figure 5B), thus underlining the shear stress effect. Indeed, HIF‐1α induction was clearly reversed when ITPP‐charged erythrocytes (RBC‐ITPP^+^) were perfused onto the hypoxic cardiomyocytes. Inhibition of HIF‐1α expression by cardiomyocytes in hypoxia was due to the oxygen release effect by ITPP‐charged erythrocytes since non‐modified erythrocytes had no influence. Moreover, this effect was not caused by ITPP itself on cardiomyocytes (Figure 5C), nor by the shear stress alone as indicated by the lack of effect of a 50 μmol/L solution of ITPP in medium, as compared to the IHP 50 μmol/L solution as control (Figure 5C).

Hypoxia was the main parameter monitoring HIF‐1α production by cardiomyocytes as shown by the control series performed in normoxia. In normoxic conditions, no effect was detected on HIF‐1α by the same treatments in flow conditions (Figure 5C).

**Figure 6:**
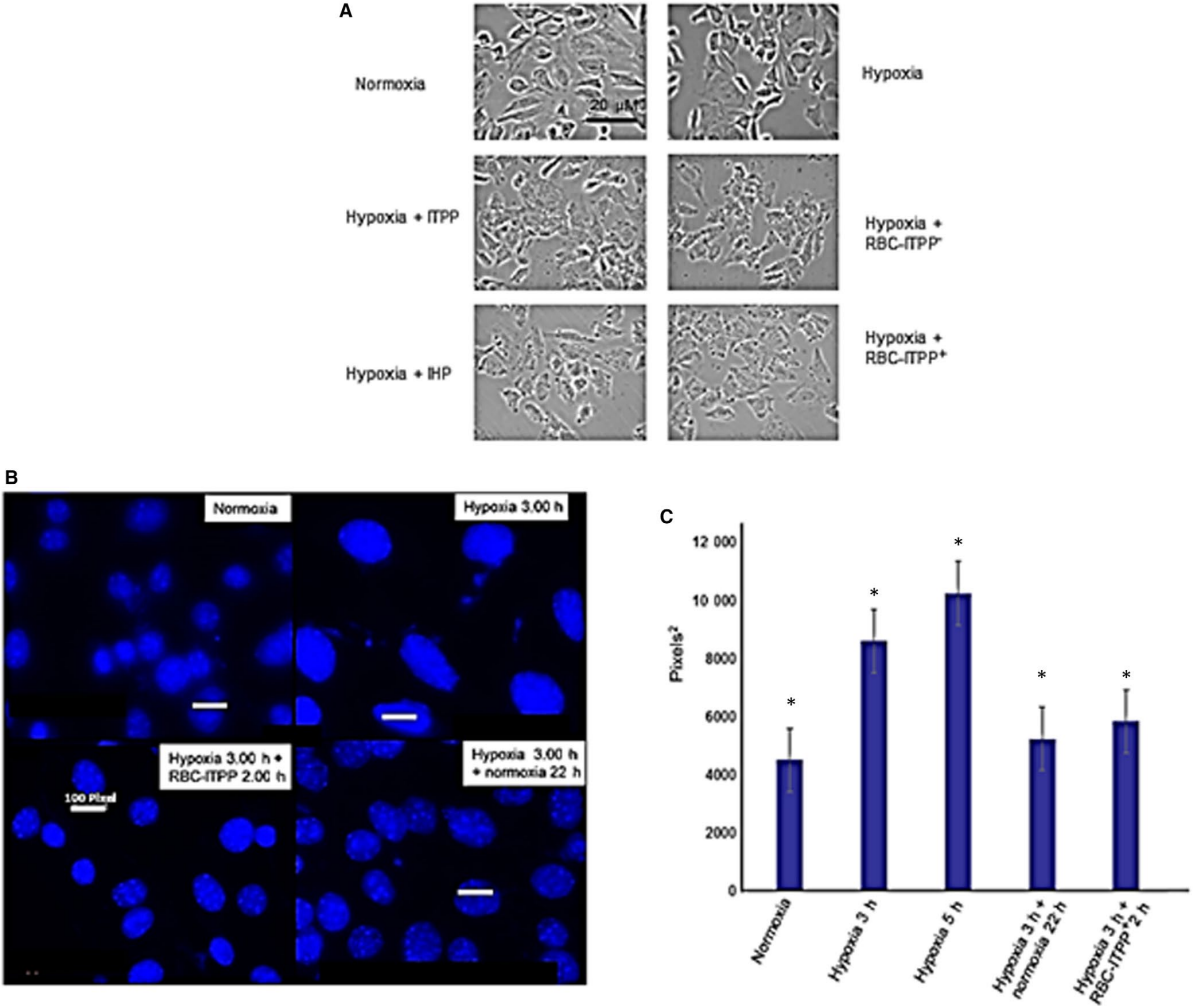
Effect of ITPP and RBC‐ITPP on cardiomyocytic cell cultures. A, ITPP and RBC‐ITPP^+^ effect of MCmCl4 cells upon hypoxia (1%, 3 h). Controls are treated by ITPP (50 μmol/L) and IHP (50 μmol/L). B, Increase of the cardiomyocytes nuclei size was observed upon hypoxia treatment (pO2 1%) for 3 h and effect of RBC‐ITPP treatment in flow (20 µL/min) for 2 h, as compared to normoxia post‐treatment for 22 h. C, Analysis of the cell evolution in hypoxia (3 and 5 h, followed by a 22 h normoxia post‐treatment or a 2 h RBC‐ITPP^+^ flow treatment). Data are mean values ± SEM from three experiments performed in triplicates

In order to rule out the possible side effects of ITPP or its control metabolite, inositol hexaphosphate (IHP), on the cardiomyocytes, we checked for their effect on cardiomyocytes growth and viability in a dose‐response assay where the cell concentration was measured after 96 hours in culture in the presence of ITPP or IHP (from 100 mmol/L). Their potential direct influence on HIF‐1α production in the same flow conditions as described above for murine erythrocytes charged by ITPP was assessed. These experiments did not reveal any effect on either the growth or the viability of the cardiomyocytes cultures as shown on Figure 6A.

As ITPP‐charged erythrocytes counteract the effects of hypoxia on cardiomyocytes and do not exert any toxicity, we addressed the question of cardiomyocytes remodelling under such treatment.

The evolution of the nuclei size was followed.^20^ Figure 6B shows that the cardiomyocytic cells, when hypoxia‐treated as above described, displayed an increased size evidenced by the number of pixels estimated for the biggest nuclei (Figure 6B, C). The size was reduced upon 2 hours of treatment by flow borne RBC‐ITPP + similarly to 20 hours normoxia post‐treatment (Figure 6B and C).

## 4 DISCUSSION

Here, we show for the first time that ITPP, a novel membrane‐permeant allosteric effector of haemoglobin that enhances the oxygen release capacity of RBC by lowering the affinity of Hb for oxygen, partially prevents post‐MI left ventricular dilation and impairment of contractility in the rat model of post‐MI heart failure, most important markers of heart failure progression and prognosis. These beneficial effects do not seem to result from improved contractile function of LV cardiomyocytes. Furthermore, ITPP administration raises no safety issues.

Moreover, our experiments in cultured murine cardiomyocytes demonstrated that when these cells were subjected to hypoxia, they exhibited overexpression of hypoxia‐inducible factor‐1 (HIF‐1α) that additionally changed its location from cytoplasmic to nuclear. This HIF‐1α overexpression could be prevented by perfusion with red blood cells charged with ITPP. Thus, we show that ITPP is a promising agent that could prevent progression of heart failure and that its effect may go beyond simple improvement of oxygen delivery to ischaemic tissues, namely involve prevention of HIF‐1 overexpression and presumably its detrimental effects.

Ischaemia (lack of adequate blood flow) and hypoxia (lack of adequate tissue oxygen) are underestimated potential mechanism of heart failure progression.^21^ Heart failure is accompanied by both myocardial hypertrophy and increased LV diastolic pressure that favour increased external compression of coronary arteries and reduction of coronary flow, especially in the subendocardial layer. Indeed, hypertrophy was shown to induce subendocardial anaerobic metabolism in dogs with aortic stenosis.^22^ Patients with dilated cardiomyopathy had reduced coronary reserve and this reduction correlated positively with fibrosis and negatively with ejection fraction.^23^ Furthermore, impaired coronary flow reserve correlated with increased serum cardiac troponin T, suggesting ongoing necrosis.^24^ Moreover, subendocardial ischaemia was associated with mitochondrial impairment and contractile dysfunction and induction of free radical production that could be prevented by anti‐oxidant interventions.^25^ Thus, large body of evidence supports the notion that ischaemia, ongoing low‐grade ischaemia or bouts of demand‐induced ischaemia triggering myocardial stunning or hibernation (), accompanies heart failure and may contribute to its progression. Moreover, in the post‐MI setting, the necrotic core is surrounded by a borderzone of live but often ischaemic tissue. With adequate oxygen delivery, it can be salvaged as healthy myocardium, but inadequate tissue oxygen may facilitate its eventual necrosis and infarct extension and further adverse remodelling. Moreover, the alleviation of ischaemia, in the setting of left ventricular dysfunction and heart failure, may be crucial for the progression.

As far as we know, we were the first to utilize a novel agent, ITPP, to improve oxygen delivery, reduce ischaemia and prevent its contribution to progression of LV dilation and impairment of function. We showed in a well‐established animal model of post‐MI heart failure, exhibiting all signs of human heart failure (progressive LV dilation, worsening of LV function) that ITPP administered over 1 month virtually halted LV dilation and impairment of EF, two most important predictors of both HF incidence after MI^26^ and prognosis in established HF.^27^ This improvement of global LV function and dilation was not accompanied by improvement of contractile function of isolated left ventricular cardiomyocytes. These results suggest that ITPP may inhibit the loss of working cardiomyocytes rather than improve their function. Indeed, cardiomyocyte loss through apoptosis and necrosis and the most recently described mechanism of programmed cell loss called necroptosis followed by replacement with fibrous tissue was considered to result in the development of LV dysfunction and progression to HF.^28^, ^29^ All processes are mediated by both death receptor and mitochondrial signalling and inadequate oxygen supply especially into cardiomyocyte subjected increased load in post‐infarction heart may promote progressive cardiomyocyte loss and subsequent impairment of LV contractile function. Another possible mechanism of beneficial ITPP effects could involve salvage of peri‐infarct borderzone, a thin layer of viable, but ischaemic myocardium.^30^

ITPP was previously used in a heart failure model of transgenic mice overexpressing Gαq.^31^ As suggested in the piglets model,^32^ in this study a single dose of 1 and 2 g/kg markedly improved maximal exercise capacity; however, no attempts of chronic ITPP administration were undertaken, nor its effects on cardiac function or remodelling were tested. However, we and others have extensively studied ITPP in oncology setting in various animal models, alone or combined with chemotherapies, and have demonstrated its potent anti‐cancer effects.^11^, ^33^ These effects at least partially were attributable to normalization of abnormal tumour vasculature through prevention of overexpression of HIF pathway that emerged as the crucial pathway for the tumour progression.^34^

In our experiments, in cultured cardiomyocytic murine cells we show that indeed ITPP is able to prevent overexpression of HIF‐1. This could be one of crucial mechanisms of its effectiveness in heart failure model in vivo, since studies confirm that HIF‐1a expression is indeed increased in myocardial samples from human failing hearts.^35^ In animal studies, overexpression of either HIF‐1α or HIF‐2α has been associated with the spontaneous development of cardiomyopathy: although HIF‐1 overexpression protected the mouse heart against consequences of MI, reduced infarct size, improved cardiac function and increased capillary density after 4 weeks of follow‐up,^36^ chronic HIF‐1a overexpression resulted in spontaneous hypertrophy and impairment of ventricular function over 8 months in mice 35 and in the model of aortic constriction was associated with impaired ventricular function.^35^ Furthermore, chronic HIF‐1a overexpression led to development of spontaneous heart failure and premature death in transgenic mouse models.^37^, ^38^ These studies suggest that while HIF‐1 overexpression can be beneficial in the short term, its long‐term effects, including metabolic reprogramming, defective angiogenesis and impairment of cardiomyocyte calcium handing, can be detrimental.^35^, ^37^

To test this hypothesis, we undertook detailed investigation of contraction and relaxation of isolated cardiomyocytes from those post‐MI rats that were treated with ITPP or placebo for 4 weeks. As we have previously demonstrated,^39^ cardiomyocytes from post‐MI hearts exhibited increased cell shortening, delayed time to peak contraction and relaxation. However, ITPP did not affect any of these parameters, suggesting that it had no effect on either contractility or relaxation. These results suggest that probably main mechanism of ITPP action in post‐MI hearts does not involve normalization of Ca^2+^ handling. However, in our model these abnormalities of Ca^2+^ handling were modest at best, so maybe in other settings, when such changes could be more pronounced, presumable effects of ITPP could be revealed.

Another very interesting aspect of our study is the mechanism in which red blood cells previously charged with ITPP, react with hypoxic cardiomyocytes resulting in oxygen release. Inherently to the red blood cells physiology, oxygen release gets more efficient when erythrocytes are delivered under flow conditions involving shear stress that more closely mimic the physiological situation.

On the other hand, ITPP itself, without red blood cells, was completely ineffective in our model, supporting the observation that it facilitates oxygen delivery by red blood cell haemoglobin rather than causing some intrinsic effects. Last but not least, ITPP did not exhibit any toxic effects, either in cultured murine cardiomyocytes, or in isolated rat cardiomyocytes or in in situ rat hearts as evidence by morphological or functional investigations.

Therefore, we can conclude that ITPP is a promising agent for the treatment and prevention of heart failure through increased tissue oxygen delivery and reduction of HIF‐1 expression may be one of its crucial potential mechanisms of action.

## CONFLICTS OF INTEREST

CN and CK are shareholders of Normoxis Inc.

## AUTHOR CONTRIBUTIONS

M. Oknińska, A. Paterek, U. Mackiewicz and M. Mączewski, conducted the in vivo experiments; B. El‐Hafny‐Rahbi, C. Crola‐Da Silva, K. Brodaczewska and C. Kieda conducted the in vitro experiments, M. Mączewski and C. Kieda designed and monitored the project and wrote the manuscript.

## Data Availability

The data that support the findings of this study are available from the corresponding author upon reasonable request.
